# Membrane Active Antimicrobial Peptides: Translating Mechanistic Insights to Design

**DOI:** 10.3389/fnins.2017.00073

**Published:** 2017-02-14

**Authors:** Jianguo Li, Jun-Jie Koh, Shouping Liu, Rajamani Lakshminarayanan, Chandra S. Verma, Roger W. Beuerman

**Affiliations:** ^1^Ocular Chemistry and Anti-Infectives, Singapore Eye Research InstituteSingapore, Singapore; ^2^Agency for Science, Technology and Research (A^*^STAR), Bioinformatics InstituteSingapore, Singapore; ^3^Duke-NUS Graduate Medical School, SRP Neuroscience and BDSingapore, Singapore; ^4^Department of Biological Sciences, National University of SingaporeSingapore, Singapore; ^5^School of Biological Sciences, Nanyang Technological UniversitySingapore, Singapore

**Keywords:** antimicrobial peptides, action mechanism, membrane, antibiotic resistance, peptide antibiotics

## Abstract

Antimicrobial peptides (AMPs) are promising next generation antibiotics that hold great potential for combating bacterial resistance. AMPs can be both bacteriostatic and bactericidal, induce rapid killing and display a lower propensity to develop resistance than do conventional antibiotics. Despite significant progress in the past 30 years, no peptide antibiotic has reached the clinic yet. Poor understanding of the action mechanisms and lack of rational design principles have been the two major obstacles that have slowed progress. Technological developments are now enabling multidisciplinary approaches including molecular dynamics simulations combined with biophysics and microbiology toward providing valuable insights into the interactions of AMPs with membranes at atomic level. This has led to increasingly robust models of the mechanisms of action of AMPs and has begun to contribute meaningfully toward the discovery of new AMPs. This review discusses the detailed action mechanisms that have been put forward, with detailed atomistic insights into how the AMPs interact with bacterial membranes. The review further discusses how this knowledge is exploited toward developing design principles for novel AMPs. Finally, the current status, associated challenges, and future directions for the development of AMP therapeutics are discussed.

## Introduction

The continued emergence of resistant pathogens world-wide, particularly among Gram-negative bacteria, has become a leading health care challenge (McKenna, 2013). Resistant strains of bacteria/fungus/yeast are regularly being found for almost every antimicrobial used clinically. On the other hand, a decline in the approval of new antibiotics has further exacerbated the problem, leading to an “antibiotic crisis” (Livermore, 2004). Persistence of the current situation is driving clinicians to use drugs that are associated with significant toxicity. In contrast to small molecule antibiotics, antimicrobial peptides (AMPs) act on the bacterial membrane which is an evolutionarily conserved component of bacterial cells (Yeaman and Yount, 2003). Bacterial membranes define the phenotype and membrane mutations would likely be costly for bacteria, therefore AMPs are less likely to induce bacterial resistance (Zasloff, 2002). Moreover, AMPs are bactericidal and they kill bacteria much more rapidly than conventional antibiotics (Zasloff, 1987; Romeo et al., 1988; Mygind et al., 2005; Marr et al., 2006; Bai et al., 2012; Deslouches et al., 2013).

There are more than 2,000 naturally occurring and synthetic AMPs (Wang G. et al., 2016). Naturally occurring AMPs, including defensins, are produced in various living organisms including humans and are part of the innate immune system (Diamond et al., 2009). In humans, these molecules are found in lymphocytes and epithelial surfaces (e.g., skin, eye, lung, and intestines etc.). For example, Paneth cells, the specialized secretory epithelial cells in the small intestine, produce high levels of AMPs (e.g., alpha-defensins, lysozyme etc.), resulting in a controlled number of bacteria in the small intestine (Ayabe et al., 2004; Bevins and Salzman, 2011). The tear fluid of the eye is also a rich source of AMPs and include the defensins, lysozyme, and cathelicidins, protecting the eyes from infections (McDermott, 2013). Other AMPs can be either synthesized ribosomally or produced non-ribosomally in bacteria and fungi during cultivation on various carbon sources (Makovitzki et al., 2006). Lipopeptides such as polymyxins are produced as metabolites in bacteria or fungi. However, these are also membrane targeting, so, in this review, the general term, AMP will be used to refer to them. In addition to their antimicrobial-activity, some AMPs have other important roles in immune regulation, inflammation, anti-cancer, sepsis, and wound healing. Indeed, some AMPs demonstrate not only antimicrobial, but also anti-inflammatory and immune regulatory activities (Sjogren, 2004; Gordon et al., 2005). The non-antimicrobial functions of AMPs are out of the scope of this review, and the interested reader can refer to relevant references (Ganz, 2003a,b; Marta Guarna et al., 2006; Van Lenten et al., 2008; McCormick and Weinberg, 2010; Shin and Jo, 2011; Li et al., 2012; Schuerholz et al., 2012; Gaspar et al., 2013; Hilchie et al., 2013; Martin et al., 2015a,b; Brandenburg et al., 2016).

AMPs are generally short peptides with <50 amino acids. Although there are more than 2,000 naturally occurring or synthetic AMPs, their lengths, sequences and 3-dimensional (3-D) conformations differ significantly, making it difficult to relate the sequence/structure to antimicrobial activity. Moreover, AMPs can undergo conformational changes upon adsorption onto the bacterial membrane and have evolved several mechanisms of action, which further complicate the analysis of a structure-activity relationship (SAR). As there is insufficient detailed knowledge of the action mechanisms in bacteria, synthetic AMPs are generally designed by trial and error (Epand and Vogel, 1999). Overcoming this gap in understanding could make antibiotic design closer to what is the common structure based approach for drug design and the action mechanism is crucial to this goal. Although the action mechanism for AMPs have been discussed in earlier reviews, most of them focus on the description of the general features of empirical models (e.g., pore-forming and carpet models), few discuss the detailed atomistic events and the dynamic processes of AMP mechanisms of action (Epand and Vogel, 1999; Shai, 2002; Guilhelmelli et al., 2013; Lee et al., 2016). Recently, the employment of molecular dynamics (MD) simulations has deepened our understanding of the underlying action mechanism at a molecular level. Based on insights at atomic level, rational molecular principles have been put forward for the development of AMP therapeutics. Our primary focus in this review is to provide a fundamental understanding of the atomistic mechanisms of various AMPs and discuss molecular principles for practical AMP design. In addition, the current status of development of AMP antibiotics as well as challenges and future prospects for AMP therapeutics are discussed.

## Modes of action of AMPs

### Structure of the bacterial membrane: the target of AMPs

**Figure 1:**
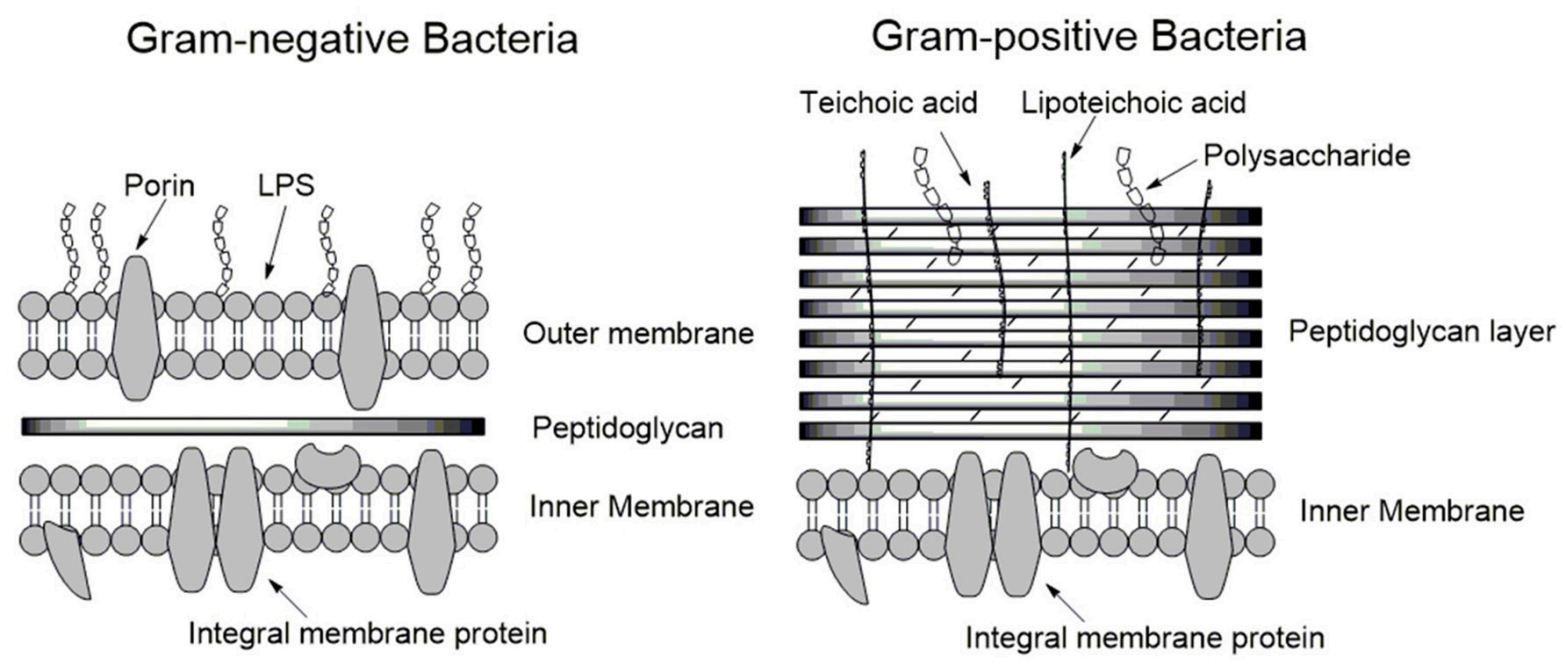
**Schematic membrane structures of Gram-positive and Gram-negative bacteria**. The cytoplasmic membranes of both types of bacteria are similar. Gram-positive bacteria have a thick layer of peptidoglycan surrounding the cytoplasmic membrane, protecting the bacterium. In contrast, the layer of peptidoglycan in Gram-negative bacteria is thin and an additional outer membrane is present. LPS forms a major part of the outer leaflet of the outer membrane/cell wall, the inner leaflet is comprised of phospholipids.

To understand the mode of action of AMPs, we begin with a discussion of the structure and physical properties of the bacterial membrane-the target of AMPs. Bacteria are broadly classified as either Gram-positive or Gram-negative, characterized by significant differences in their cell envelopes (Figure 1). The inner or cytoplasmic membranes of both bacteria groups are similar. However, the outer cell envelopes are significantly different. In Gram-positive bacteria, there is a layer of crosslinked peptidoglycan decorated with negatively charged teichoic acid surrounding the cytoplasmic membrane, forming a thick matrix which maintains the rigidity of the bacterial cell. Nano-sized pores penetrate into the peptidoglycan layers, allowing AMPs to diffuse through (Meroueh et al., 2006). In contrast, the peptidoglycan layer in Gram-negative bacteria is much thinner and less cross-linked. In addition, Gram-negative bacteria have an additional outer membrane outside the peptidoglycan layer. The inner leaflet consists purely of phosphate lipids while the outer leaflet is primarily a coat of lipopolysaccharide (LPS; Ruiz et al., 2006). LPS molecules are decorated with a high number of negatively charged phosphate groups that are engaged in salt-bridges with divalent cations (e.g., Ca^2+^ and Mg^2+^), resulting in an electrostatic network (Nikaido, 2003). This electrostatic region serves as a primary barrier to most hydrophobic antibiotics, resulting in low permeability. Therefore, the details as to how AMPs penetrate into Gram-positive and Gram-negative bacteria must vary in their atomistic interactions. In the case of Gram-positive bacteria, AMPs need to diffuse across the peptidoglycan matrix first and then act on the cytoplasmic membrane. In contrast, killing Gram-negative bacteria involves perturbation or disruption of both outer and cytoplasmic membranes. Inability to permeabilize or disrupt the outer membrane results in the loss of antimicrobial activity. Daptomycin is able to disrupt the cytoplasmic membrane, but not able to permeabilize/disrupt the outer membrane of Gram-negative bacteria. As such, it is highly active against Gram-positive bacteria such as methicillin-resistant *Staphylococcus aureus* (MRSA), but has no activity against Gram-negative bacteria (Tally and DeBruin, 2000).

### Parameters affecting AMP movement to the cytoplasmic membrane

The cytoplasmic membrane (also called as the inner membrane) of Gram-positive and Gram-negative bacteria are comprised of a mixture of zwitterionic and anionic phospholipids, such as phosphatidylethanolamine (POPE), phosphatidylglycerol (POPG), and cardiolipin (CL). Models of action for AMPs acting on the cytoplasmic membrane include pore formation (e.g., barrel stave or toroidal pores) and carpet mechanism (Brogden, 2005; Melo et al., 2009). For an AMP to disrupt the cytoplasmic membrane, the AMP molecules must first accumulate on the membrane surface up to a critical concentration. However, diffusion barriers in either the outer membrane or the periplasmic space affect their partition onto the membrane. This is a more direct path for Gram-positive bacteria as the AMPs only need to diffuse through nano-sized pores in the peptidoglycan, which in most cases is not a rate-limiting step (Vollmer et al., 2008). In fact, the peptidoglycan layer can facilitate AMP accumulation on the surface of the cytoplasmic membrane due to favorable interactions between teichoic acid and cationic AMPs (Malanovic and Lohner, 2016).

**Figure 2:**
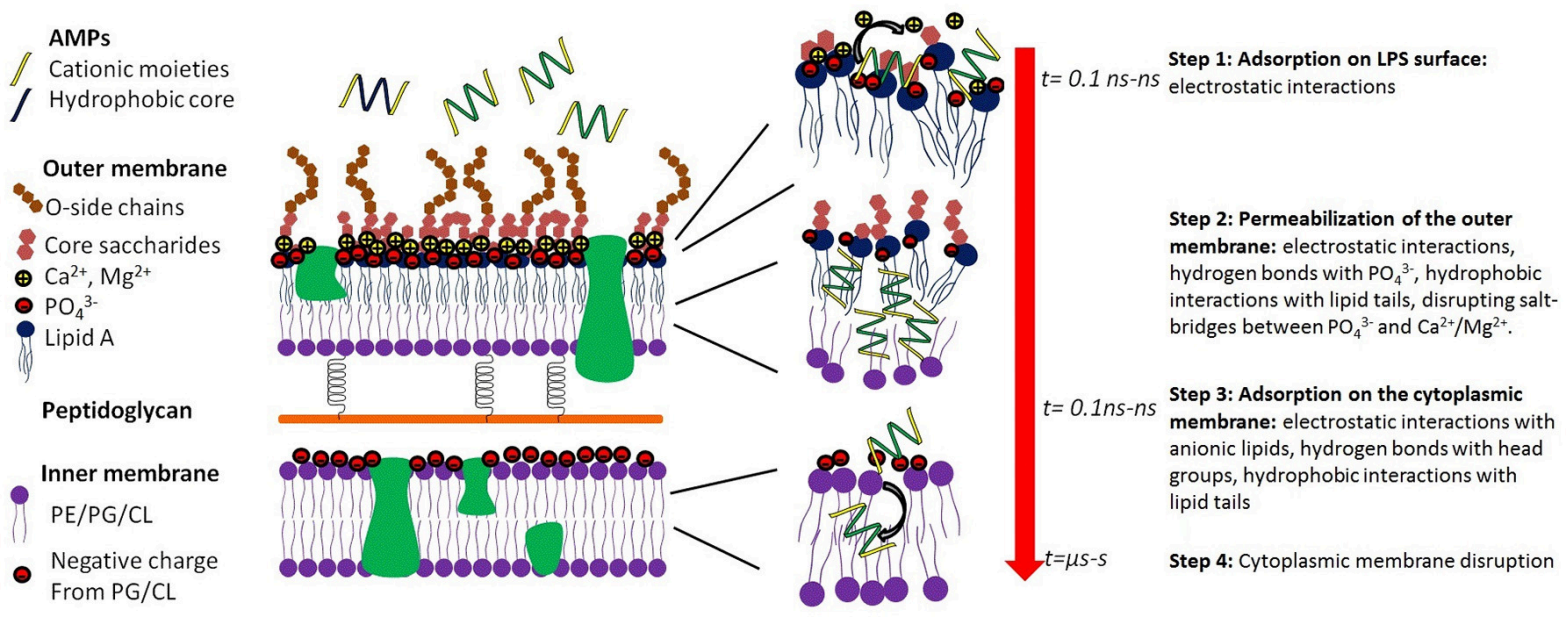
**Key molecular events of the interaction of an AMP which results in killing Gram-negative baceria**. Step 1, adsorption of the AMP to the surface of the outer membrane, which is very rapid (*t* = ns, nanosec) and is driven by electrostatic interactions. Step 2, permeabilization of the outer membrane, which is mediated by complex interactions including electrostatic interactions, hydrogen bonds, and hydrophobic interactions. In step 3, the AMP diffuses through the periplasmic space and reaches the surface of the inner membrane. Step 4 is the disruption of the cytoplasmic membrane, loss of the transmembrane potential, influx of water, and killing of the bacteria.

In the case of Gram-negative pathogens, AMPs need to permeabilize or disrupt both the outer and cytoplasmic membranes, resulting in a two-step process (Figure 2; Schwechheimer and Kuehn, 2015). Although the outer membrane of Gram-negative pathogens significantly modulates the antimicrobial activity of AMPs, in most cases, the inner membrane is the rate-limiting step. For example, polymyxin B has strong antimicrobial activity against Gram-negative pathogens due to its ability to disrupt both the outer and the cytoplasmic membranes. However, removing the lipid tail resulting in polymyxin B nonapeptide, and only the outer membrane is permeabilized. As a result, the nonapeptide also loses antimicrobial activity (Ofek et al., 1994).

The detailed mode of interactions between AMPs and the outer membrane of Gram-negative bacteria is poorly understood due to the complex structure of the LPS molecules, which consists of a lipid A component, an inner and an outer core portion and a sugar portion (Figure 2). The initial event is the adsorption onto the outer membrane surface, which can occur within tens of nanoseconds and is largely mediated by electrostatic interactions between cationic AMPs and anionic LPS molecules. Reduction in the electrostatic driving force significantly affects the partition of AMPs to the outer membrane and may result in the loss of antimicrobial activity. For example, modification of the phosphate groups on the LPS molecules such as deacylation, hydroxylation, or addition of phosphoethanolamine endows bacteria resistance to colistin (polymyxin E; Olaitan et al., 2014). Upon adsorption onto the outer membrane, AMPs form hydrogen bonds with the phosphate groups, disrupting the salt-bridges between phosphate groups and divalent cations and destabilizing the outer membrane. In addition to the electrostatic forces, the hydrophobic moieties of an AMP can also interact with the lipid tails of the LPS molecule, further destabilizing the close packing of the outer membrane. After the AMP permeabilizes/disrupts the outer membrane, it diffuses inward to the periplasmic space and adsorbs onto the surface of the cytoplasmic membrane. Once a critical surface concentration on the cytoplasmic membrane is reached, the AMP induces significant perturbations and disorganizations of the cytoplasmic membrane, resulting in loss of the transmembrane potential, and eventual death of the bacterial cell.

Characteristics of the interactions of AMP with the outer and cytoplasmic membranes include: length of the AMP sequence, the total and density of cationic charges, the total number of hydrogen bonds donors and the 3-D conformation of the AMP in solution and at the membrane (Sitaram and Nagaraj, 1999; Bürck et al., 2008). The AMP structures that have been determined experimentally and deposited into the protein data bank provide little information for SAR as these structures are either in solution or in crystals rather than in the membrane environment. Due to the short sequences of most AMPs, their structures are sensitive to their environment. By comparing the CD spectrum of AMPs in solution and in the presence of liposomes, a large number of AMPs (e.g., melittin, magainin) have been found to undergo conformational transitions from random conformations in solution to helical or beta-sheet conformations on the membrane surface (Ramamoorthy et al., 2006; Hartings et al., 2008). For example, melittin adopts a helical conformation and protegrin adopts a beta-sheet conformation upon adsorption onto the membrane surface (Blazyk et al., 2001; Lu et al., 2005; Rausch et al., 2005, 2007; Dong et al., 2012). *In silico* modeling further reveals that both the alpha-helical and beta-sheet conformations are amphiphilic, with the cationic residues interacting with the anionic head groups and the hydrophobic residues penetrating into the lipid tail region of the membrane. Hence the electrostatic and hydrophobic interactions are two driving forces that steer an AMP toward and into the bacterial membrane.

### Early events in AMP disruption of the cytoplasmic membrane

After adsorption onto the membrane surface, AMPs can induce a variety of membrane perturbations within tens of nanoseconds (Li et al., 2013). For most AMPs, an early event is the formation of hydrogen bonds between the basic residues (e.g., arginine and lysine) and the phosphate groups of the lipids. At physiological conditions, both lysine and arginine are hydrogen bond donors. However, arginine can form more stable bidentate hydrogen bonds with phosphate groups. Hydrophobic residues can further penetrate and disorganize the lipid tail region of the membrane. As more AMP molecules accumulate at the membrane-water interface, the membrane becomes thin, as observed for most AMPs (Sato and Feix, 2006; Ye et al., 2010). Moreover, as the total volume of the membrane is roughly constant, membrane thinning results in lateral expansion, affecting the mechanical properties of the membrane. For example, with the increase in the area per lipid, the surface tension increases and the bending modulus decreases dramatically, implying membrane deformation (Szleifer et al., 1988; Stevens, 2004). The expansion of the membrane also results in reduction in the packing of the lipid molecules leading to the formation of a large number of cavities, significantly reducing the translocation free energy of water molecules across the lipid tail region. As a result, a large number of water translocations occur, and the membrane becomes leaky with the collapse of the transmembrane potential and additional membrane dysfunction, such as inhibition of ATP production and loss of proton motive force (Dimroth et al., 2000) and rapid death of the bacterium.

Some AMPs, particularly the highly cationic ones can induce lipid re-arrangements and the formation of lipid rafts. B2088, an 18 residue peptide dimer with a high content of basic residues (12 positive charges), once adsorbed on the bacterial membrane, was found to recruit anionic lipids surrounding the peptide, resulting in micro-domains rich in anionic lipids in the outer leaflet of the membrane (Bai et al., 2012). Due to the binding to AMPs, lipid molecules surrounding AMP molecules display slow diffusivity, which not only affects the fluidity and structure of the membrane, but also results in significant tension, particularly along the domain boundaries (Guo et al., 2011). Clustering of anionic lipids in model bacterial membranes was found for other peptides as well (Oreopoulos et al., 2010; Polyansky et al., 2010; Wadhwani et al., 2012; Scheinpflug et al., 2015). Some AMPs were found to even induce flip-flop of anionic lipids from the inner leaflet to the outer leaflet, resulting in highly negatively charged outer leaflet and less negatively charged inner leaflet (Qian and Heller, 2011). This asymmetric distribution of charged lipids further destroys the membrane potential, resulting in membrane destabilization.

Membrane geometry as measured by the curvature is a fundamental property denoting stability with its basis in lipid organization and protein inclusions. Wong and coworkers suggested that negative Gaussian curvature is a prerequisite topology for the formation of membrane pores (Schmidt et al., 2010, 2011; Schmidt and Wong, 2013). It was found that POPE has the highest tendency to form a negative Gaussian curvature (“saddle-splay”) because of its small head group with respect to its large lipid tails (Yang et al., 2007; Schmidt et al., 2011). Interestingly, bacterial membranes consist of a high percentage of POPE while human membranes are mainly composed of POPC, implying that the preference of AMPS for POPE-rich membranes contributes significantly to the selectivity of AMPs. MD simulation studies have also shown that arginine residues can induce higher negative Gaussian curvature than lysine residues. The guanidinium group of arginine can form bidentate hydrogen bonds and coordinate two phosphate groups at <0.5 nm, while the distance is 0.7 nm for lysine (Schmidt et al., 2012a,b; Wu et al., 2013). Wu et al. employed both coarse-grained and atomistic models to simulate the interactions of poly-arginine and poly-lysine with model bacterial membranes (Wu et al., 2013). Their results revealed the different interactions of arginine and lysine residues with lipid molecules. The guanidinium group of arginine can simultaneously interact with both phosphate and glycerol groups, and thus induce greater negative Gaussian curvatures than lysine residues, as the latter can only engage interactions with phosphate groups. Wong and coworkers also found that incorporation of hydrophobic moieties further reinforces negative Gaussian curvature, which is consistent with the antimicrobial activity of various arginine-rich AMPs. For example, a series (RW)n peptides, which consists of only arginine (R) and tryptophan (W), display high antimicrobial activity (Strøm et al., 2002, 2003; Chan et al., 2006). In particular, an analog of (RW)n peptide LTX-109 has entered into clinical trials (Isaksson et al., 2011b).

### Molecular models of pore formation

**Figure 3:**
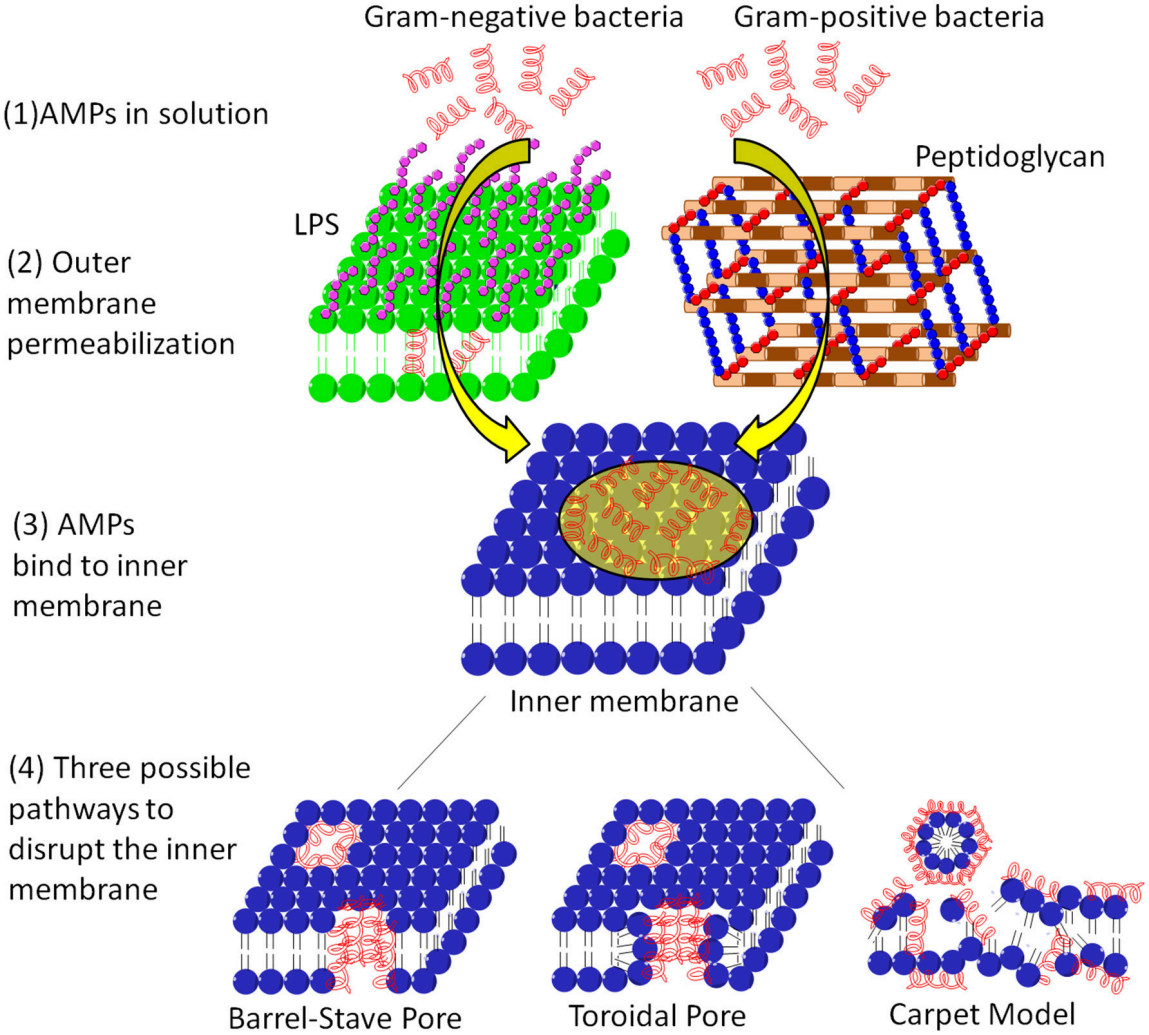
**The membrane systems of Gram-positive and Gram-negative bacteria and the action mechanisms of AMPs on the cytoplasmic membrane**. For Gram negative bacteria, the AMP needs to permeabilize the outer membrane before reach the cytoplasmic membrane, whereas for Gram positive bacteria, the AMP only needs to diffuse through the peptidoglycan layer via nano-sized pores. After adsorption onto the cytoplasmic membrane, the AMP can induce membrane pores such as barrel-stave pore and toroidal pore, or defragment the membrane.

A number of AMPs form membrane pores (Table 1). The timescale of pore formation ranges from microseconds to seconds. Membrane pores result in the loss of membrane potential and rapid release of intracellular components and death. Depending on the geometry of the pores as well as the interactions of AMPs with the pores, pores can be described by the barrel-stave or the toroidal model (Figure 3). The formation of barrel-stave pores is driven by hydrophobic match. In this model, the membrane does not display significant curvature and the hydration of the membrane remains unchanged. In the case of the toroidal model, as the AMP molecules penetrate deeper into the membrane, the head groups of the lipids are dragged into the lipid tail region to form toroidal shaped pores while the lipid tails are packed away from the surface of the pore, resulting in significant lipid disorder, and membrane curvature change (Sengupta et al., 2008). As a result, toroidal pores are also accompanied by enhanced membrane hydration, as evidenced by significant water penetration into the membrane (Sengupta et al., 2008; Manna and Mukhopadhyay, 2009). In toroidal pores, AMPs primarily interact with the pores electrostatically since the surface of toroidal pores are covered by the phosphate head groups and the AMP molecules have less hydrophobic contact with the lipid tails. In contrast, in barrel stave pores, both electrostatic and hydrophobic interactions are important since the AMP molecules interact with both the head groups and the lipid tails (Mihajlovic and Lazaridis, 2010; Bertelsen et al., 2012).

**Table 1:** **Representative AMPs and their modes of action**.

**AMPs**	**Sequence**	**Mechanism**	**References**
Maculatin 1.1	GLFVGVLAKVAAHVVPAIAEHF	Pore	Dye leakage experiment and MD simulations (Chen and Mark, 2011; Sani et al., 2013)
Caerin 1.1	GLLSVLGSVAKHVLPHVVPVIAEHL	Pore	MD simulations (Chen and Mark, 2011)
Cateslytin	RSMRLSFRARGYGFR	Pore	MD simulations and Patch-clamp experiment (Jean-François et al., 2008)
Gramicidin A	VGALAVVVWLWLWLW	Pore	NMR (Urry, 1971; Ketchem et al., 1993)
Alamethicin	PAAAAQAVAGLAPVAAEQ	Barrel stave pore	Ion conductance experiment and statistical analysis (Boheim, 1974; Laver, 1994)
Magainin H2	IIKKFLHSIWKFGKAFVGEIMNI	Pore	Ion conductance experiment and MD simulations (Matsuzaki, 1998; Leontiadou et al., 2006)
Melittin	GIGAVLKVLTTGLPALISWIKRKRQQ	Toroidal pore	Dye leakage and grazing-Angle X-Ray Anomalous Diffraction and MD simulations (Yang et al., 2001; Sengupta et al., 2008; Lee et al., 2013; Leveritt et al., 2015)
Protegrin 1	RGGRLCYCRRRFCVCVGR	Pore	Ion conductance experiment (Sokolov et al., 1999)
LL-37	LLGDFFRKSKEKIGKEFKRIVQRIKDFLRNLVPRTES	Toroidal pore	NMR (Henzler Wildman et al., 2003)
Indolicidin	ILPWKWPWWPWRR	Pore	Ion conductance experiment (Falla et al., 1996)
Pardaxin 1	GFFALIPKIISSPLFKTLLSAVGSALSSSGEQE	Barrel stave pore	NMR (Porcelli et al., 2004; Ramamoorthy et al., 2010)
MSI peptide	GIGKFLHSAKKFGKAFVGEIMNS	Carpet	NMR (Lee et al., 2015)
Citropin 1.1	GLFDVIKKVASVIGGL	Carpet	MD simulations (Chen and Mark, 2011)
Aurein 1.2	GLFDIIKKIAESF	Carpet	Quartz crystal microbalance with dissipation, vesicle dye leakage and atomic force microscopy experiments and MD simulations (Chen and Mark, 2011; Fernandez et al., 2012)
B2088	(RGRKVVRR)_2_KK	Carpet	MD simulations (Li et al., 2013)
PL-5	Ac-KWKSFLKTFKS-A-AKTVLHTALKAISS-amide		In clinical trials. ProteLight-Pharmaceuticalal, 2016^a^

a *http://www.protelight.com/english/Content.asp?Action=ArticleShow&Columns=26&Category=27&Articleid=80*.

Although induction of membrane pores is a general mechanism for a large number of AMPs, the morphology of these membrane pores can be quite diverse in terms of pore diameter, lipid conformation surrounding the pore, life time of the pore (e.g., transient pore or permanent pore) and the number of AMP molecules required to stabilize the pore. Regarding the shape of the pore, AMPs such as alamethicin, dermcidin, and pardaxin induce barrel stave pores (Laver, 1994; Porcelli et al., 2004; Song et al., 2013), while most other AMPs induce toroidal pores (Matsuzaki, 1998; Sokolov et al., 1999; Yang et al., 2001; Henzler Wildman et al., 2003; Sengupta et al., 2008; Lee et al., 2013). In terms of the pore size, magainin induces small toroidal pores of ~ 2–3 nm in diameter that only allow water and small intracellular molecules to leak out (Takeshima et al., 2003; Brogden, 2005), while lacticin Q and protegrin can form much larger pores of 4.6 and 9 nm in diameter, respectively, allowing the leakage of much large intracellular molecules (Yoneyama et al., 2009; Lam et al., 2012). In addition, the pore size depends on various factors such as the lipid composition and the peptide/lipid ratio. At nanomolar concentrations, melittin can only induce transient pores. As the melittin concentration increases beyond a critical value (e.g., peptide/lipid ratio of 1/45), it induces stable membrane pores of 2.5–3 nm in diameter, and the pore size further increases with the peptide/lipid ratio (Terwilliger et al., 1982; Matsuzaki et al., 1997; Lee et al., 2013). Another well-studied AMP, maculatin can also form an ensemble of structurally diverse pores (Wang Y. et al., 2016). Moreover, the formation of membrane pores depends on the lipid composition of the membrane as well. Evidence for this lies in the observation that most AMPs induce membrane pores at a much higher concentration in mammalian membranes than in bacterial membranes. The former mainly consists of zwitterionic lipids (e.g., POPC) and cholesterol, which are closely packed and thus have a low tendency to form membrane pores. In contrast, bacterial membranes consist of high percentage of POPE, which has a small head group and thus has higher tendency to form negative Gaussian curvatures, as discussed above (Schmidt and Wong, 2013).

Biophysical methods used to study the pore structure as well as AMP conformations in the pores, include small angle x-ray scattering (SAXS), oriented circular dichroism (OCD, solid-state NMR, quartz crystal microbalance (QCM; Yang et al., 2007; Kwon et al., 2013; Wang et al., 2015; Bürck et al., 2016). However, these methods typically give the early or final states of the AMP-membrane complex, e.g., the static structures of AMPs and membrane pores. The dynamics of the pore formation process is missing because the intermediate states of pore formation are either in non-equilibrium or metastable states and thus have life times that are too short to be observed. To obtain dynamic information on pore formation, MD simulations using atomistic and coarse-grained models have been carried out (Edit et al., 2007; Kirsch and Böckmann, 2016). Due to the complexity of the pore formation process, the sub-microsecond time scales that MD simulations can access and the fact that pore formation is a rare event, it is still difficult to observe the complete process of pore formation in conventional MD simulations. Nevertheless, Leontiadou et al. were the first to successfully simulate the process of toroidal pore formation by magainin (Leontiadou et al., 2006). In the simulations, the toroidal pore displays higher disorder than the normal toroidal pore model, reflecting the dynamic nature of pores in membranes. In another simulation from the same group, similar effects were observed for melittin. One or two melittin molecules were observed to line within the pore axis, while other peptide molecules located at the two ends of the pore (Sengupta et al., 2008). Removing the positive charges of melittin molecules failed to induce membrane pores, revealing the importance of electrostatic interactions. Interestingly, in both simulations, peptides lose their helical conformations when binding to the membrane pores, suggesting that the helical conformation is not necessary for stabilization of the membrane pores, at least for the case of toroidal pores. Coarse-grained MD simulations were also performed for melittin and transient toroidal pores were observed at high peptide/lipid ratios (Santo et al., 2013). It was shown that typically 3–5 melittin molecules are involved in each pore and similar to the atomistic simulations, not all the melittin molecules span across the membrane. The difference in the pores between atomistic and coarse-grained simulations suggests diverse morphologies of melittin induced membrane pores. These MD simulations, although only carried out at a micro-second time scale, nevertheless provide valuable molecular insights into the action mechanism of AMPs.

### Carpet mechanism

Not all AMPs induce membrane pores. Some AMPs adsorb onto the membrane surface and orient parallel to the membrane. As their surface concentrations reach a critical value, these AMPs disrupt the integrity of the super molecular structure of the membrane via membrane fragmentation, a process called carpet mechanism or detergent model (Figure 3; Gazit et al., 1996). Some examples of AMPs with carpet mechanism are listed in Table 1. In the carpet model, the outer leaflet is covered by a high surface concentration of AMP molecules, while the inner leaflet is free of AMP binding. The large imbalance of charge and surface tension across the membrane eventually leads to catastrophic collapse of membrane integrity and leakage of the cytoplasmic contents, ions, and biomolecules. In contrast, in the pore forming mechanism, only intracellular molecules smaller than the pore can exit. The size of the released molecules can be used to distinguish the pore forming mechanism from a carpet mechanism.

In the carpet mechanism, the complete picture of membrane collapse is not well-understood because (i) the process is highly non-equilibrium; (ii) multiple pathways of membrane lysis exist, making it difficult to be detected in biophysical experiments. However, at a peptide concentration lower than the critical concentration, the membrane undergoes some intrinsic perturbations such as membrane thinning, lateral expansion, clustering of anionic lipids, and membrane deformation, and high surface tension. Interestingly, these membrane perturbations can be used to correlate to the subsequent membrane lysis. For example, Wimley proposed an interfacial activity model to relate these membrane perturbations to the membrane activity of an AMP (Wimley, 2010). In the surface activity model, the membrane activity of an AMP is a function of the surface activity, which depends on two elements: (i) the partition of an AMP to the surface of the bacterial membrane and (ii) the ability to induce membrane perturbations. As the former is mostly driven by electrostatic interactions, AMPs with greater charge are more likely to achieve high concentrations on the membrane surface. The latter depends on many factors such as the 3-D conformation of the AMP, the physical properties of the AMP, and the actual interactions of the AMP with the lipids. Given the fact that the membrane is amphiphilic in nature, AMPs with facial amphiphilicity display high affinity for the membrane. The destabilization of the hydrophobic-water interface is critical for subsequent membrane lysis. In addition, membrane perturbations can be further enhanced via AMP aggregation, a mechanism similar to the toxic effects of an amyloid peptide (Milov et al., 2009; Di Scala et al., 2016). For example, LL-37, HAL-2, and the C-terminal segment of human beta-defensin 3, can achieve high surface concentration via self-aggregation upon adsorption onto the surface (Bai et al., 2009; Wang et al., 2014; Bonucci et al., 2015). The self-aggregation of maculatin 1.1, a membrane-active antimicrobial peptide (AMP) from the Australian tree frog leads to deeper penetration into the membrane core and significant change of curvature of the membrane (Bond et al., 2008). The self-aggregation of AMPs leads to an enhanced local concentration of AMPs around the membrane and has been used to design multimeric AMPs (Arnusch et al., 2007; Bai et al., 2012; Shin et al., 2013; Lakshminarayanan et al., 2014; Koh et al., 2015a).

## Methods for AMP design

Although research on AMP development has been highly active in the past years, few molecules have entered into the market. For an AMP to be used as a therapeutic agent, it needs to possess several druggable properties: (i) high antimicrobial activity; (ii) low toxicity to the mammalian membrane; (iii) high proteolytic stability; (iv) low serum binding; and (v) low cost. Most AMPs have minimum inhibitory concentration (MIC) values <10 μg/ml, while conventional antibiotics may have MICs in the range of 1–2 μg/ml or even sub μg/ml range, suggesting that significant effort is still needed to design new AMPs with higher activity. Various approaches have been employed to design new AMPs with low MIC, high stability, and low toxicity. These methods include the time-consuming mutation based empirical method, statistically based bioinformatics methods, and more sophisticated mechanism based methods such as MD simulations and biophysical experiments. Recently, multi-disciplinary approaches combining computational predictions, biophysical characterizations and biological validations have proved to be more promising (Bai et al., 2012; Koh et al., 2015a; Lakshminarayanan et al., 2016). This section discusses various experimental and computational methods that have been used for AMP design. Clearly, if the details of the membrane interactions were known design of an appropriate AMP would be facilitated.

### Mutation based empirical methods

Early attempts to design AMPs were largely carried out by trial and error due to poor understanding of the action mechanism. Many designed AMPs are actually based on the modifications of existing natural AMPs. A number of approaches can be tried to re-engineer a naturally occurring AMP, such as sequence shuffling and alanine scanning. Sequence shuffling, in which the positions of the amino acids in the sequence are changed can be the simple sequence reversal or via a combinatorial library. Since sequence shuffling does not change the hydrophobicity and the net positive charges, it has been used to optimize the antimicrobial activity of specific AMPs (Monroc et al., 2006; Cherkasov et al., 2009). The technique of alanine scanning mutates each residue or a group of residues to alanine and the antimicrobial activity is monitored for improvement. Alanine scanning has been widely used in AMP design and has proved to be very useful in identifying residues critical for antimicrobial activity (Grieco et al., 2011; Hänchen et al., 2013).

### Statistically based bioinformatics methods

AMP databases enhance computer aided AMP design (Fjell et al., 2007; Thomas et al., 2010; Wang G. et al., 2016). Various bioinformatics tools have been developed, including simple statistical modeling, SARs, neural networks, and machine learning. In general, these bioinformatics based tools need a database with known antimicrobial activity of certain peptides as the training set. By using different bioinformatics algorithms, key structural, and biophysical features are extracted from the training set and can be used to predict the antimicrobial activity of unknown peptides, which is referred to as the test set. For example, Mishra and Wang employed database filtering technology to determine the key parameters of AMPs (e.g., amino acid composition, peptide hydrophobic content, and net charge) and subsequently used these parameters for the ab initio design of potent AMPs against MRSA (Mishra and Wang, 2012). Besides the AMP database, design principles can also be obtained from other sources, such as genomes and proteomes (Li et al., 2007; Brand et al., 2012; Kim et al., 2016).

### Mechanism based methods

Although the bioinformatics methods are simple and fast, they are still black boxes, as there is little information that reveals the action mechanisms. A more powerful and rational method would be to design new AMPs based on the mode of interaction with the membrane. To this purpose, detailed understanding of the action mechanisms of existing AMPs is required. MD simulation is a powerful method that can give atomistic information regarding the interactions of AMPs with bacterial membranes. If the time scale of the simulation is long enough, membrane disruption or pore formation can be directly observed (Wang Y. et al., 2016). MD simulations have been previously used to decipher the action mechanism of AMPs, and recently have been successfully applied to the design of new AMPs and antimicrobial peptidomimetics (Tsai et al., 2009; Tew et al., 2010a; Li et al., 2013, 2015; John Fox et al., 2016). For example, Bai et al. performed atomistic MD simulations of a short cationic AMP B1088 and found that the charge density plays an important role in its interactions with bacterial membrane mimics (Bai et al., 2009). Based on this information, they subsequently designed a covalent peptide dimer B2088 and a tetramer B4010 which demonstrated enhanced antimicrobial activity and proteolytic stability (Li et al., 2013; Lakshminarayanan et al., 2014). By using coarse-grained MD simulations, Tew et al. successfully designed a number of synthetic mimics of AMPs with high membrane selectivity (Tew et al., 2010a). One of their compounds brilacidin is currently under phase II clinical trials.

NMR can also provide mechanistic details for improved AMP design and in fact complements MD simulations. Similar to MD simulations, NMR, particularly solid state NMR, can provide information regarding the 3-D conformation of the peptide as well as the mode of interactions with model lipid membranes (Strandberg and Ulrich, 2004; Su et al., 2010). These include identification of the biophysical properties of critical residues that mediate the interactions with the membrane. For instance, Saravanan et al. used NMR combined with other biophysical experiments to design tryptophan and arginine rich decamer peptides and potent antimicrobial activity and low toxicity of the decamer peptide were found to arise from an optimal ratio between the positive charges and hydrophobicity (Saravanan et al., 2014). Later the same group used NMR to further design β-boomerang lipo-peptides that neutralized LPS (Mohanram and Bhattacharjya, 2014). Jeong et al. used NMR experiments not only to design a series of LPcin analogs with potent antimicrobial activities, but also elucidated the 3-D structure of a peptide-membrane complex (Jeong et al., 2016).

### Principles for practical design of AMPs

Based on the methods discussed above, some general design principles have been proposed which can be directly used to guide AMP design and are briefly discussed below.

#### Amphiphilicity

Amphiphilicity is perhaps the most striking feature of AMPs, including facial amphiphilicity (Vandenburg et al., 2002), bola-amphiphilicity (Ali, 2007), radial amphiphilicity (Xiong et al., 2015), etc., all with at least one cationic moiety and one hydrophobic moiety. Considering the amphiphilic nature of membranes, amphiphilic peptides are expected to have high membrane affinity. Facially amphiphilic AMPs are usually helical with one side being cationic and the other side being hydrophobic. When adsorbed onto the bacterial membrane, these AMPs locate at the membrane surface, with the cationic face interacting with the head groups and the hydrophobic face penetrating into the lipid tail region, resulting in significant perturbations at the membrane-water interface. On the other hand, bola-amphiphilic peptides are expected to adopt transmembrane conformations, driven by hydrophobic match. In such a case, the two cationic moieties interact with the two head group regions, while the hydrophobic moiety interacts with the lipid tails. When several bola-amphiphilic peptide molecules oligomerize in the bacterial membrane, membrane pores can be formed (Matile et al., 2011).

#### Peptide crosslinking

As both the pore forming mechanism and the carpet mechanism depend on the concentration of the peptide on the bacterial membrane surface, various methods have been proposed to enhance the surface concentration of AMPs. An effective way to enhance surface concentration of AMPs is through peptide self-aggregation, which leads to more effective membrane disruption compared to the monomeric peptide. For example, LL-37 self-aggregates on the bacterial membrane leading to the formation of toroidal pores (Bonucci et al., 2015). However, if the peptide is highly cationic, the self-aggregation is inhibited due to electrostatic repulsion between AMP molecules. In such a case, covalent linking can be used to generate covalent peptide aggregates, known as multimeric peptides. As discussed above, the covalent peptide dimer B2088 and tetramer B4010 displayed much higher antimicrobial activity than the peptide monomeric unit comprising these peptides (Bai et al., 2012; Lakshminarayanan et al., 2014, 2016).

#### Role of Arg

Most AMPs are cationic as a result of a high percentage of basic residues, Lys or Arg. Although both carry positive charges at neutral pH, the p*K*a values of Arg (12.45) and Lys (10.5) are different, and this will affect their protonation states in the membrane environment. Theoretical calculations found that Arg will retain its protonation state in the lipid tail region of the membrane, while Lys becomes deprotonated in the bilayer center (Yoo and Cui, 2008, 2010; Gleason et al., 2013). Due to the high p*K*a and multi-dentate hydrogen bonding property of Arg, Arg-rich peptides are thought to have stronger interactions with membranes. For example, a twin-arginine motif was found to assist peptide translocation and polyarginine itself is an efficient cell penetrating peptide (Chaddock et al., 1995; Bechara and Sagan, 2013). Studies also showed that Arg can induce more negative Gaussian curvatures than Lys due to its bidentate hydrogen bonding with PO4 groups (Schmidt et al., 2010, 2011; Schmidt and Wong, 2013; Wu et al., 2013). Accordingly, various Arg-rich AMPs have been designed. For example, (RW)n peptides display excellent antimicrobial activity (Liu et al., 2007). The side chain of Arg residue, the guanidine group was found to greatly enhance the action of antimicrobial peptidomimetics compared to the side chain of Lys residue (Andreev et al., 2014). Although many studies have shown the preference of Arg over Lys in terms of antimicrobial activity, some peptides prefer Lys over Arg residues. For example, arginine modified polymyxin B displayed reduced antimicrobial activity, suggesting that there appears not to be a general rule for selective preference of Arg and Lys by AMPs (Rabanal et al., 2015).

#### Peptide truncation

Most of the classical antimicrobial peptides are fairly large and expensive to synthesize. This has led to the design of ultra-short peptides, with only 3–4 amino acids. The sequence of the peptidic moiety and the length of the hydrophobic moiety appear to determine the spectrum of antimicrobial activities. Despite their short lengths, their modes of action involves permeation and disintegration of the membrane organization, similar to that of many classical AMPs (Makovitzki et al., 2006) As electrostatic interactions with the bacterial membrane are still involved, almost all ultrashort peptides designed and synthesized to date contain cationic amino acids, such as arginine and lysine (Findlay et al., 2012; Domalaon et al., 2014; Mishra et al., 2015) For instance, KYR is an amino acid sequence of bovine hemoglobin alpha chain, which was part of the longer amino acid sequence that was obtained from hydrolysing the hemoglobin alpha chain. KYR is one of the shortest AMPs known (Nasompag et al., 2015). Most of the ultrashort peptides are conjugated with a fatty acid tail to provide additional hydrophobicity to kill bacteria efficiently (Makovitzki et al., 2006)

#### Incorporating unnatural amino acids

The synthetic AMPs are not restricted to the 20 natural amino acids. Instead, they can incorporate various unnatural amino acids or have additional chemical modification. The direct advantage of AMPs containing unnatural amino acids is their high proteolytic stability. More importantly, as AMPs require a delicate balance of cationic and hydrophobic groups, chemical modifications enables easy fine-tuning of the hydrophobic balance. The commonly used approach for chemical modification includes use of more hydrophobic amino acids, lipid, and aromatic modifications. For example, lipid modifications of the above mentioned peptide dimer B2088 results in C8-B2088, which demonstrated enhanced antimicrobial activity (Koh et al., 2015a). Similarly, modification of the Phe residue with an additional benzene ring significantly enhances the antimicrobial activity of a short peptide FRFR-NH2, while maintaining its low toxicity to mammalian membranes (Lau et al., 2015). Multiple modifications have been used together to achieve high activity. LTX-109, a short synthetic AMP with both lipid and aromatic modifications, has been in clinical trials (Saravolatz et al., 2012b). Recently, a pharmacophore model has been proposed for the design of short AMP mimetics with the sequence of RXR, where X is a hydrophobic scaffold (Li et al., 2015). Derivatives of the pharmacophore model have shown excellent activity against resistant pathogens, low toxicity to mammalian membranes, and extremely high stability (Koh et al., 2015b).

### Current status: examples of AMPs/peptidomimetics in clinical trials

In the past 30 years, continuous efforts have been made to develop AMPs as clinically useful antimicrobials due to their advantages over conventional antibiotics such as a rapid bacterial killing, good selectivity toward the bacterial membrane, and a low propensity to give rise to bacterial resistance (Bai et al., 2012). However, to date, no designed AMP antibiotics have yet reached the clinic. Nevertheless, as described below, a number of AMPs and AMP derivatives are already at the pre-clinical stage and in clinical trials.

PL-5 is an α-helical AMP with a sequence of Ac-K-W-K-S-F-L-K-T-F-K-S-A-A-K-T-V-L-H-T-A-L-K-A-I-S-S-amide, where Ac = N-acetyl and amide = C-terminal amide. PL-5 is developed by ProteLight Pharmaceuticals and has recently obtained approval from the China Food and Drug Administration (CFDA) to enter clinical trials for skin infection in the year 2016. It is noteworthy that PL-5 is the first AMP to enter the clinical stage in China. PL-5 is a low toxicity and highly potent AMP against a broad spectrum of drug-resistant bacteria. In addition, PL-5 is able to synergize with conventional antibiotics to improve antibacterial activity *in vitro* and *in vivo* against both Gram-positive and Gram-negative bacteria. This may help prevent or delay the emergence of antibiotic resistance (Feng et al., 2015).

POL7080 is a synthetic cyclic peptide derived from protegrin I. POL7080 is active against Gram-negative bacteria and works by inhibiting a homolog of the β-barrel protein LptD. LptD is an outer-membrane protein widely distributed in Gram-negative bacteria that functions in the assembly of LPS in the outer leaflet of the outer membrane (Braun and Silhavy, 2002) LptD is involved in the outer-membrane biogenesis of lipopolysaccharide. Significantly, POL7080 is highly active on a broad panel of clinical isolates including multi-drug resistant Pseudomonas with outstanding *in vivo* efficacy in septicemia, lung and thigh infection models (Polyphor) POL7080 is developed by Polyphor Ltd and has competed a phase I clinical trial with its partner Roche. POL7080 has also completed a phase-II trial in 20 patients with exacerbation of non-cystic fibrosis bronchiectasis in 2015 (Butler et al., 2017). To date, the structure of POL7080 has not been revealed.

DPK-060 is a cationic peptide that has recently completed a Phase II study of topical application for atopic dermatitis. DPK-060 is a broad spectrum cationic peptide active against both Gram-positive and Gram-negative bacteria. Similar to other AMPs, DPK-060 is also membrane targeting (Harvey et al., 2015). DPK-060 is developed by Pergamum AB. The results from a Phase II clinical trial of DPK-060 in outer ear infections showed a statistically significant improvement in a 10-day cure rate compared to placebo and that DPK-060 is safe and tolerable (Lee et al., 2015) However, no recent reports of DPK-060 development have been forthcoming. Pergamum AB has also developed LL-37, a human cathelicidin subunit. Human cathelicidin is synthesized by numerous cells as an inactive precursor, hCAP18/LL-37. It consists of a highly conserved N-terminal signal sequence, a conserved cathelin domain, and a small antimicrobial C-terminal domain. The small antimicrobial C-terminal domain is known as LL-37 (Vandamme et al., 2012). The LL-37 domain is released by cleaving the proteolytic hCAP18/LL-37 precursor. This domain exhibits antimicrobial activities against both Gram-positive and Gram-negative bacteria (Overhage et al., 2008; Barlow et al., 2011) LL-37 has a peptide sequence of LLGDFFRKSKEKIGKEFKRIVQRIKDFLRNLVPRT. LL-37 is developed for treatment of chronic leg ulcers. The clinical phase results show that LL-37 has a significantly improved healing rate compared to placebo (Lee et al., 2015).

Innate Defense Regulators (IDRs) are a novel class of synthetic peptides that enhance the control of microbial infections. IDRs do not impact the adaptive immune system and do not interfere with chemotherapy, radiation therapy or antibiotic treatments. SGX942 contains the active ingredient dusquetide (also referred to as SGX-94). Dusquetide is a fully synthetic, 5-amino acid peptide derived from Indolicidin with high aqueous solubility and stability (Soligenix) SGX94, has broad-spectrum activity against Gram-negative and Gram-positive bacterial infections caused by intracellular or extracellular bacteria and also complements the actions of standard of care antibiotics (North et al., 2016). Since SGX-94 acts through host pathways to provide both broad-spectrum anti-infective capability as well as control of inflammation, IDRs are unlikely to be impacted by resistance mechanisms. It also offers potential clinical advantages in the fight against emerging and antibiotic resistant bacterial infections (North et al., 2016) SGX-942 was first developed by Inimex and is currently being pursued in a Phase-II trial by Soligenix as treatment for oral mucositis (Butler et al., 2017). SGX942 has previously demonstrated safety and tolerability in a double-blind, placebo-controlled, healthy volunteer Phase I clinical trial. SGX942 has been awarded Fast Track designation from the FDA for the treatment of oral mucositis as a result of radiation and/or chemotherapy treatment in head and neck cancer patients.

There are also several AMPs that were not approved by FDA or failed at an earlier development stage, such as Iseganan, Omiganan, and XMP-629 and Locilex (Ahmad et al., 2012). Dipexium acknowledged that Locilex did not meet the primary clinical endpoint of superiority vs. placebo or “vehicle” namely the cream without its active ingredient of pexiganan. In order to overcome the major limitations of AMPs such as systemic toxicity and proteolytic instability, the development of small-molecule-based membrane-targeting antimicrobials that maintain the essential key characteristics of AMPs, has received considerable attention (Lohan and Bisht, 2013). Peptidomimetics are a new generation of small-molecule antimicrobials that mimic the structure and antibacterial action of AMPs. Design of peptidomimetics involves the introduction of amide bond isosteres or peptide backbone modifications via non-natural side chains to mimic a peptide structure or function (Niu et al., 2012; Lohan and Bisht, 2013).

**Figure 4:**
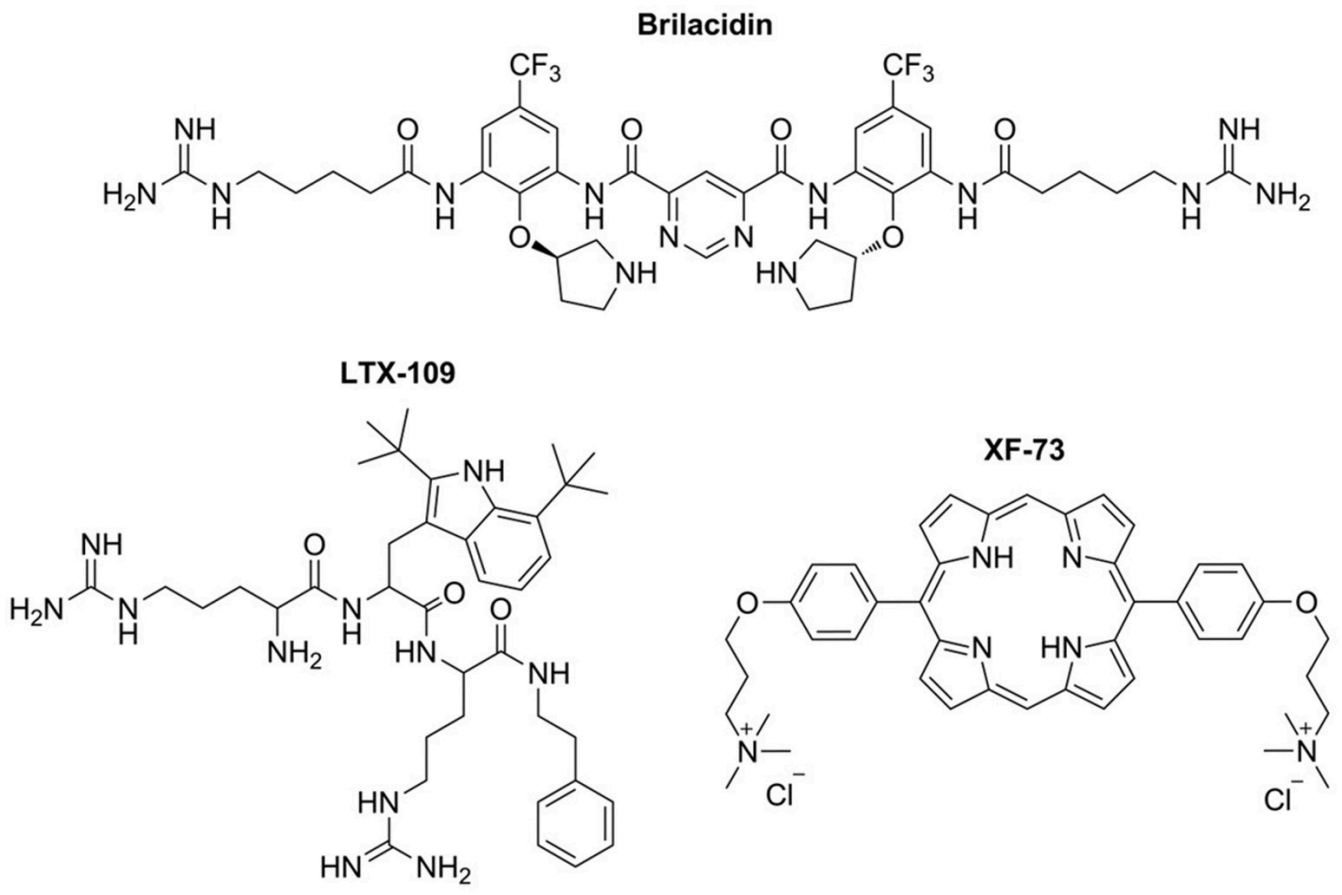
**Structures of AMP mimetics in clinical studies**. All the three molecules contain a large hydrophobic moiety and two cationic moieties, forming a cationic-hydrophobic-cationic motif, and mimicking the interactions of AMPs with the bacterial membrane.

Brilacidin is a small-molecule arylamide mimic of AMPs that shows potent antimicrobial activity against a wide range of drug-susceptible and multidrug-resistant Gram-negative and Gram-positive bacteria (Tew et al., 2002, 2010b; Liu et al., 2004) Brilacidin has a planar, conformationally restrained scaffold with four positive guanadinyl and pyridinyl substitutions and two trufluoromethane hydrophobic substitutions (Figure 4). Brilacidin was first developed by Polymedix Inc. and purchased by Cellceutix corp. in September 2013 (Butler et al., 2017). Brilacidin has completed phase IIa and phase IIb trials for the treatment of acute *S. aureus* skin and skin structure infections. Compared to daptomycin, the results show no serious adverse effects and the efficacy is similar to daptomycin across all brilacidin treatment groups in 215 patients Similar to other AMPs, brilacidin is a membrane targeting antimicrobial. It causes membrane disruption and shows efficacy in a MRSA keratitis model when applied topically. At 0.5% solution, brilacidin shows minimal irritation and is equally efficacious as vancomycin (Kowalski et al., 2016) In addition to brilacidin, Cellceutix is also developing CTIX-1278 (structure not revealed), a defensin mimetic-compound, against the drug resistant superbug *Klebsiella pneumoniae*. CTIX-1278 is efficacious in a thigh burden study using a mouse model. The results are encouraging as CTIX-1278 shows similar efficacy compared to carbapenem.

LTX-109 is developed by Lytix Biopharma, which focus on topical treatment of skin infections and nasal eradication of staphylococcus. LTX 109 is a synthetic antimicrobial peptidomimetic, which has completed phase 2 trials for the treatment of impetigo in the year 2014 and uncomplicated skin and skin structure infection (uSSSI) in the year 2011 (Butler et al., 2017). LTX-109 has the chemical structure Arg-Tbt-Arg-NH-EtPh (Figure 4). Arg provides the cationic charge and the tertiary butyl group is important to increase the hydrophobicity. In general, LTX-109 is active against a broad range of bacteria including *E. coli* and *S. aureus* (Isaksson et al., 2011a) Significantly, LTX-109 is also active against a panel of drug-resistant Gram-positive bacteria, such as MRSA, vancomycin-intermediate resistant, daptomycin resistant, and linezolid-resistant strains (Saravolatz et al., 2012a) LTX-109 kills bacteria via the membrane targeting mechanism.

Exeporfinium chloride (XF-73) is a synthetic dicationic porphyrin derivative being developed by Destiny Pharma (Brighton, UK) that has been evaluated in phase-I/II trials for the prevention of post-surgical staphylococcal nasal infections (Figure 4; Butler et al., 2017). XF-73 has completed 5 Phase I/IIa clinical studies in Europe/US. XF-73 is a photosensitizer that has broad-spectrum antimicrobial activities against Gram-positive, Gram-negative and *Candida albicans* (Farrell et al., 2010; Gonzales et al., 2013). XF-73 exhibits potent, non-lytic, bactericidal activity against *S. aureus*. Similar to AMPs, interaction of XF-73 with the cytoplasmic membrane is lethal to *S. aureus*, leading to release of intracellular components and bacterial cell death (Ooi et al., 2009). In addition, XF-73 showed no drug resistance emergence from four common MRSA strains tested in a multi-step (55 passage) resistance study, as a ≤ 4-fold increase in MIC against the strains tested (Farrell et al., 2011). On 05 September 2016, Destiny Pharma announced that XF-73 can be delivered safely and is well-tolerated in a two-stage US clinical trial using intra-nasally applied exeporfinium chloride gels (DMID contract number HHSN27220800026C). In addition, no drug was detected in the bloodstream (Destiny Pharma, accessed on 12 January 2017).

Peptides have appeared in a wide range of applications in other clinical areas (Fosgerau and Hoffmann, 2015). For instance, thymalfasin, a short peptide with 28 amino acids, has been used in clinics for its immune regulatory function (Sjogren, 2004). With more and more emerging strategies to design new generation AMPs with improved efficacy, safety, and tolerability, we believe that peptide antibiotic still offer enormous growth potential to reach the clinic in the near future.

## Challenges and future perspectives

### Current AMP development: challenges and solutions

AMP development has been an active research area in the past 30 years, but only recently has there been a positive outlook for commercial success. There are challenges that limit the design of potent AMPs, such as the poor understanding of the target-drug interaction and the lack of rational design principles. Besides activity, issues such as toxicity, serum binding, stability, and product cost are also practical considerations. Solutions to overcome these limitations have been proposed and have become hotspots of current AMP research and development. A side benefit of AMP research is that emphasis has changed from screening unknowns in a microbiology setting to defining the target bringing antibiotic development closer to conventional structure based drug development.

Toxicity of AMPs can occur at different levels, including membrane toxicity, cellular toxicity, and systemic toxicity. Membrane selectivity is a widely accepted parameter to characterize *in vitro* membrane toxicity and is defined as the ratio of HC50/MIC, where HC50 is the concentration needed to cause 50% hemolysis of human red blood cells. As stated earlier in this review molecular charge of bacterial membranes and membranes of human cells differ so that AMPs with higher positive charges show enhanced affinity for the bacterial membrane, resulting in higher antimicrobial activity (lower MIC) and conversely less toxicity to human cells (Zelezetsky and Tossi, 2006). Moreover, it is also proposed that AMP hydrophobicity can affect human membrane toxicity, a factor useful in the design of branched lipo-peptides with minimal toxicity (Koh et al., 2015a).

Cellular/metabolic toxicity and systemic toxicity are more difficult to predict as the underlying mechanisms are complex. Cellular toxicity refers to single cell toxicity, which can be measured for human cells using MTT assays, LDH release, and ATP synthesis (Fotakis and Timbrell, 2006). Systemic toxicity can arise from various effects such as activation of transcription factors, binding to macromolecular receptors in the body, alteration of metabolic pathways, and triggering immune response, making it challenging to predict. For example, polymyxin B, the last resort for the treatment of multi-drug resistant Gram-negative bacteria, although safe at the membrane level, can cause significant nephro- and neurotoxicity (Falagas and Kasiakou, 2006). To address and predict the issues surrounding systemic toxicity, several strategies have been employed. Computational toxicology uses machine learning algorithms to predict toxic outcomes (Valerio, 2009). Another strategy is via formulation (Carmona-Ribeiro and de Melo Carrasco, 2014). For example, Gramicidin, a topical AMP is effective against many Gram-positive bacteria, but has significant hemolysis. However, incorporating gramicidin in a dioctadecyldimethylammonium bromide (DODAB) bilayer not only results in reduced toxicity, but also leads to broader antimicrobial activity against both *E. coli* and *S. aureus* (Ragioto et al., 2014).

AMPs consisting of all natural amino acids may need to enhance their proteolytic stability. This limitation may not be a serious problem for topical applications, but results in significantly reduced half-life in systemic applications. Various approaches can be used to enhance the proteolytic stability of AMPs. The direct way is to mutate key amino acids at the cleavage site to D amino acids or similar analogs. For example, arginine can be replaced by D-Arg or homoarginine, while lysine can be replaced by D-Lys or ornithine. However, the effect of L-to-D mutation on the antimicrobial activity needs to be re-evaluated, although in most cases the L-to-D mutation does not alter the antimicrobial activity significantly (Hong et al., 1999; Berthold et al., 2013; Carmona et al., 2013). In addition, chemical modifications as discussed in Section Principles for Practical Design of AMPs, such as incorporation of unnatural amino acids and cross-linking can function to improve peptide stability.

Cationic AMPs tend to display high affinity for serum proteins, decreasing the available concentration of drug; however, at the same time this is a general issue with most antibiotics. For example, it was shown that AMPs can interact with drug site II of albumin via hydrophobic interactions (Sivertsen et al., 2014). In addition, the cationic residues of most cationic AMPs make them good substrates for the chymotrypsin family of endoproteases (Perona and Craik, 1997). The strong protein binding property significantly reduces the effective concentration of the AMP available to combat bacteria (Svenson et al., 2007). Moreover, host cells can also interfere with the activity of AMPs. Starr et al. pointed out that interactions with host cells can lead to significant loss of activity *in vivo*, in a way very similar to the effects of serum protein binding (Starr et al., 2016). To develop AMPs with less binding to serum proteins/host cells, detailed PK/PD studies are required. Compared to small molecule antibiotics, AMPs may be more expensive to produce; however, this limitation can be overcome by the use of synthetic biology (Cameron et al., 2014). Using genetically engineered microbial fermentation, large amounts of recombinant peptides can be produced. For example, a fusion protein containing the antimicrobial sequence at its C-terminus was successfully expressed in *E. Coli*., and subsequent cleavage released AMP P2 (Haught et al., 1998).

### Future perspectives

Importantly, AMPs probably represent the best option for the treatment of multi-drug resistant infections. Considerable effort has been expended in this area with progress and a number of AMP/peptidomimetics are in different phases of clinical trials. Since the MIC values for most AMPs are still higher than many conventional antibiotics, the primary task is to improve the antimicrobial activity, reduce the toxicity, and improve delivery efficiency. Another promising area is the design of membrane active peptidomimetics to mimic the action of existing AMPs, which can be achieved by chemical modification of existing AMPs or using unnatural amino acids. Compared to AMPs, peptidomimetics greatly expand the molecular space of membrane active antimicrobials and have the advantages of high proteolytic stability and optimizing the hydrophobicity. However, this needs to be coupled with the more detailed understanding of the molecular and atomistic interactions between AMPs/peptidomimetics and the molecular complex of the Gram-negative membrane system. Computer aided drug design, particularly the mechanism based *in silico* design approach such as MD simulations has a great potential to help overcome some of these limitations. When combined with other methods in a multi-disciplinary setting, translation of fundamental knowledge to practical clinical therapeutics can be greatly accelerated. This approach should also be activated to overcome the AMP resistant strains such as the recently appeared colistin (also known as polymyxin E) resistant strains (Fernández et al., 2013; Olaitan et al., 2014; Li et al., 2015). The advantages of combining the *in silico* simulations and NMR is that the approach is adaptable to the challenge of bacteria with modified LPS structure. If successful a new age of antibiotics could be forthcoming with less resistance, longer clinical utility and greater opportunities for special purpose design of antibiotics and other antimicrobials.

## Author contributions

JL, JK, and SL wrote the drafted the manuscript; RL, CV, and RB modified the manuscript. All authors discussed and contributed to the manuscript.

### Conflict of interest statement

The authors declare that the research was conducted in the absence of any commercial or financial relationships that could be construed as a potential conflict of interest.

## Abbreviations

AMP: Antimicrobial peptide
POPE: Phosphatidylethanolamine
POPG: Phosphatidylglycerol
POPC: Phosphatidylcholine
POPS: Phosphatidylserine
CL: Cardiolipin
LPS: Lipopolysaccharide
SAR: Structure-activity relationship
CD: Circular dichroism
MD: Molecular dynamics
MRSA: methicillin-resistant *Staphylococcus aureus*
MIC: minimum inhibitory concentration.
